# Generalized Doppler effect for high-accuracy frequency shift measurement

**DOI:** 10.1038/s41377-026-02259-9

**Published:** 2026-04-13

**Authors:** Yanxiang Zhang, Dexin Ba, Yang Yang, Yongkang Dong

**Affiliations:** 1 National Key Laboratory of Laser Spatial Information, Harbin Institute of Technology, Harbin, China; 2 Zhengzhou Advanced Research Institute, Harbin Institute of Technology, Zhengzhou, China

**Keywords:** Optical metrology, Optical sensors

## Abstract

Laser Doppler effect enables a wide range of precision measurements. However, its traditional implementations, including linear, rotational, and vectorial forms, have historically been treated as isolated phenomena, and meanwhile, their accuracy is fundamentally limited by the achievable frequency shift magnitude due to single controllable parameter. Here, we report a generalized Doppler effect that overcomes these limitations and enhances metrological accuracy. Such effect arises when tailored vectorially polarized dual-vortex fields derived from spin-orbit coupling interact with moving scatterers. In doing so, we observe four simultaneous spectral signatures in a single measurement, including conventional Doppler signal (DS), Doppler polarization signal (DPS), and two novel Doppler polarization-vortex signals (DPVSs). Crucially, the advanced DPVSs with coupled polarization (*m*) and orbital angular momentum (*ℓ*), produce amplified frequency shifts that enhance relative measurement accuracy by factors scaling as *κ*_*1*_(1 + |*m*|/*ℓ*) and *κ*_*2*_(1 + *ℓ*/|*m*|), compared with conventional DS and DPS schemes. Furthermore, directional ambiguity inherent to these shifts can be resolved via phase analysis of either initial polarization offset or analyzer angle difference. Our generalized framework not merely unifies previous Doppler formulations but offers a potential pathway to substantially improved Doppler metrology, enabling unprecedented accuracy in high-resolution fluid vorticity mapping, quantitative hemodynamic monitoring, and next-generation LiDAR systems.

## Abstract (web summary)

This work reported a generalized Doppler effect that unified existing linear, rotational and vectorial frameworks, enabling unprecedented high-accuracy Doppler-shifted measurement.

## Graphical abstract

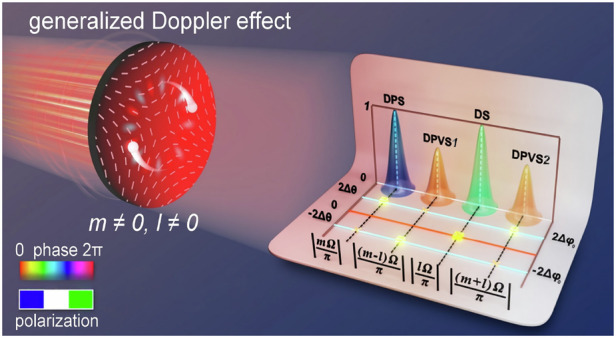

## Introduction

Laser Doppler effect, manifested as a frequency shift in light reflected or scattered from moving objects, enables accuracy measurement of velocity and vibration information^1,2^. This unique capability, combined with its advantages of rapid response, high sensitivity, broad dynamic range, and non-contact operation, makes it indispensable for diverse applications, such as wind-tunnel diagnostics^3^, speed monitoring^4^, and meteorological sensing^5^, etc. In particular, the flourishing developments of extreme shock physics, clinical medicine, and autonomous navigation have driven the need for high-speed, non-intrusive measurement^6–8^, which match well with the advantages of laser Doppler metrology. Consequently, laser Doppler metrology has become a focus of research, with recent advances significantly expanding its capabilities. Nevertheless, current laser Doppler metrology face three critical challenges: (ⅰ) conventional linear Doppler schemes can only detect radial Doppler shift, completely missing transverse component; (ⅱ) while capable of quantifying the magnitude of the frequency shift, cannot distinguish red/blue shift; and (ⅲ) existing Doppler schemes still operate within a fragmented framework without a unified theory, and their measurement accuracy remains limited by the achievable frequency-shifted magnitude. Therefore, it is urgently needed to develop unified Doppler scheme capable of overcoming these limitations and enabling high-accuracy direction-sensitive detection of Doppler-shifted vector.

**Fig. 1:**
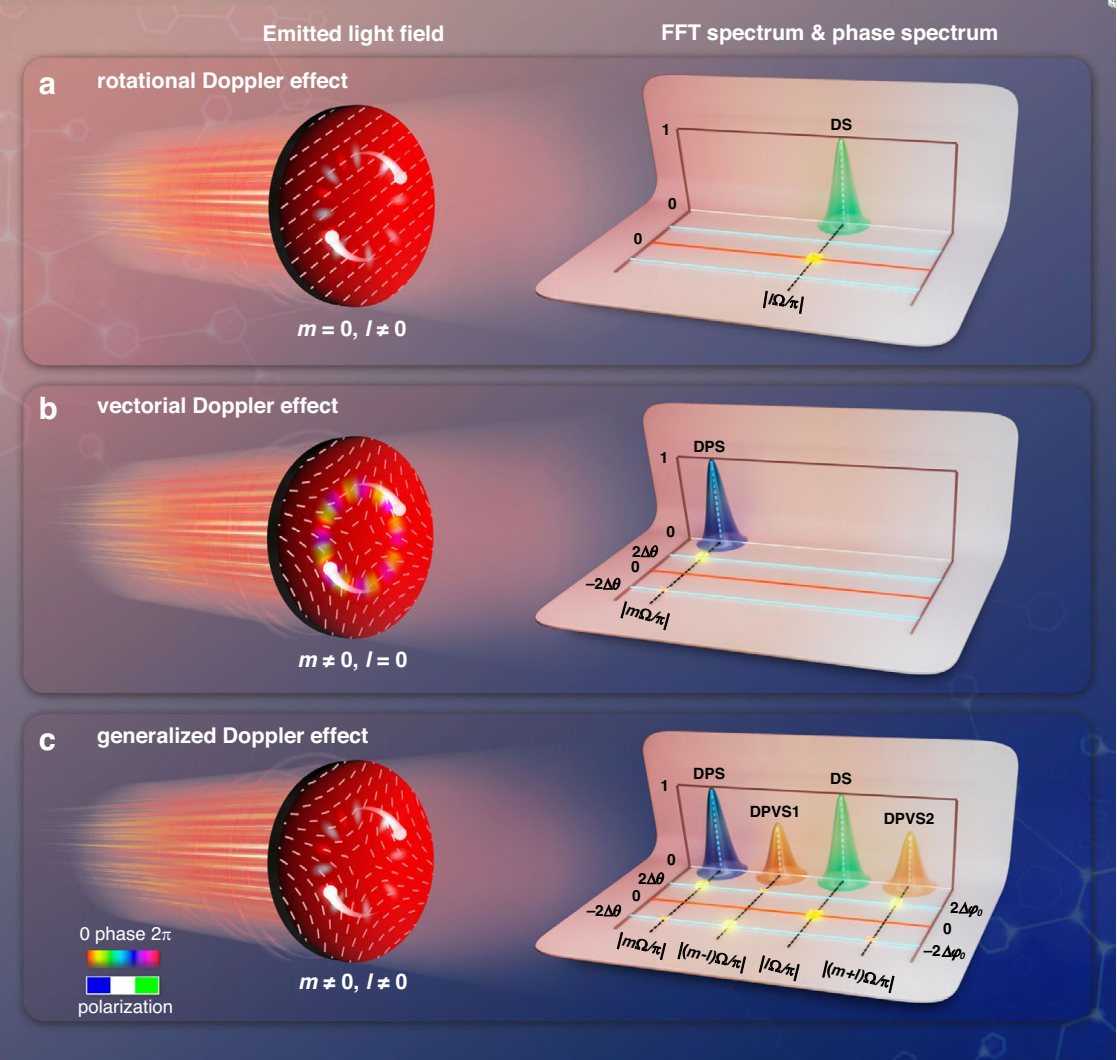
Conventional Doppler effect versus generalized Doppler effect. **a** Rotational Doppler effect: phase-conjugated vortex field (*m* = 0, *ℓ* ≠ 0) interacting with a rotational particle (angular velocity, Ω). The FFT spectrum shows a single DS peak at *Δf*_*DS*_ = |*ℓΩ/π*|, while the phase spectrum fails to discriminate red/blue shifts (yellow cross/square: red/bule shift induced by ±*Ω*)^14–16^. **b** Vectorial Doppler effect: vectorially polarized field (*m* ≠ 0, *ℓ* = 0) interacting with the same particle. The FFT spectrum shows a single DPS peak at *Δf*_*DPS*_ = |*mΩ/π*|, with red/blue shifts unambiguously resolved in the phase spectrum via linear polarization angle difference *Δθ*^22–24^. **c** Generalized Doppler effect: VPDVF (*m* ≠ 0, *ℓ* ≠ 0) interaction yields multiple spectral signatures, including DS, DPS, and DPVS1, DPVS2, with peaks at *Δf*_*DS*_ = |*ℓΩ/π*|, *Δf*_*DPS*_ = |*mΩ/π*|, *Δf*_*DPVS1*_ = |(*m-ℓ)Ω/π*|, *Δf*_*DPVS2*_ = |(*m* + *ℓ)Ω/π*|, respectively. The red/blue shifts in the phase spectrum are unambiguously resolved via both linear polarization angle difference *Δθ* and initial polarization-angle offset *Δφ*_*0*_ for DPS, DPVS1, DPVS2, whereas the DS component remains directionally indistinguishable

Unlike conventional linear Doppler effect, which is restricted to measuring merely longitudinal Doppler shift due to its Poynting vector parallel to optical axis, rotational Doppler effect overcomes this limitation by leveraging orbital angular momentum (OAM)-carrying light fields with tilted Poynting vector^9–22^. As illustrated in Fig. 1a, rotational Doppler metrology based on beating between phase-conjugate OAM modes (±|*ℓ*〉) allows accuracy extraction of the frequency-shifted magnitude |*ℓ*Ω/π| via the fast Fourier transform (FFT) spectrum. However, since the frequency spectrum does not convey the sign of the shift, the rotation direction remains ambiguous. Conversely, the vectorial Doppler effect (Fig. 1b) overcomes this limitation by exploiting spin–orbit interactions between vectorially structured light and moving targets, encoding both the magnitude and direction of the shift (±*m*Ω/π) directly in the output polarization state^23–25^. This approach resolves the first two challenges, enabling detection of transverse Doppler shifts and unambiguous determination of red/blue shift without the need for additional modulation or interferometric setups^26–29^. Despite these significant advances, the absence of a unified theoretical framework and the limited accuracy imposed by restricted OAM and polarization orders have not yet been addressed.

In this context, we theoretically predict and experimentally demonstrate a versatile generalized Doppler effect, arising from interactions between developed vectorially polarized dual-vortex fields (VPDVFs) and moving scatterers. Unlike conventional schemes (Fig. 1a, b), our approach yields a generalized Doppler spectrum that simultaneously reveals three distinct signal types: traditional Doppler signals (DS), Doppler polarization signals (DPS) and a newly identified class of Doppler polarization-vortex signals (DPVSs) (Fig. 1c). The VPDVFs, which carry both polarization order *m* and orbital angular momentum order *ℓ*, are generated via spin–orbit coupling. They are fully characterized by Stokes parameters for polarization and eigenmode decomposition for OAM. Their OAM power spectrum exhibits pronounced spin–orbit coupling, in stark contrast to traditional vortex beams^30–32^. Using a beat-frequency detection scheme integrated with polarization-decoupling techniques, we extract the generalized Doppler signal (GDS). Crucially, its red/blue shift can be unambiguously discriminated by measuring either linear polarization-angle difference (*Δθ*) or the initial polarization-angle offset (*Δϕ*_*0*_), the latter previously unexplored. Moreover, by tuning *m* and *ℓ*, the GDS can be seamlessly collapsed to linear/rotational or vectorial Doppler effects or to the new vector-vortex Doppler effect. Most notably, one of the DPVSs achieve unprecedented accuracy in frequency-shifted measurement: *κ*_*1*_(1 + |*m*|/*ℓ*)-fold superior to conventional DS metrology and *κ*_*2*_(1 + *ℓ*/|*m*|)-fold beyond existing DPS approaches. This breakthrough effectively delivers a high-accuracy pathway with a larger frequency shift than previous Doppler methodologies. Our results, therefore, establish a unified and highly accurate framework for advanced Doppler measurements.

## Results

### Concept and principle

Figure 1c schematically presents the conceptual framework for observing generalized Doppler effect and enhancing Doppler-shifted measurement accuracy. To achieve this, we firstly construct and characterize a novel type of detection light source, referred as VPDVFs (see Supplementary Note 1). Specifically, the tailored light fields carrying simultaneous polarization order *m* and dual OAM orders *ℓ₁* = -*ℓ₂* = *ℓ* are generated based upon spin-orbit coupling, and then characterized by Stokes parameters for the polarization as well as characterized by modal decomposition for the OAM (see Methods). In cylindrical coordinates (*r*, *φ*, *z* = 0), the electronic field of such structured fields can be expressed as,

1 $$\vec{E}(r,\varphi )=\sqrt{2}{A}_{0}(r)({e}^{i{\ell }_{1}\varphi }+{e}^{i{\ell }_{2}\varphi })\left[\begin{array}{l}\cos (m\varphi +{\varphi }_{0})\\ -\,\sin (m\varphi +{\varphi }_{0})\end{array}\right]$$

where *A*_*0*_ is a spatial position *r*-related amplitude. *φ*_*0*_ is the initial polarization angle.

Subsequently, such an engineered field is directly launched onto an isotropic particle moving at an angular velocity *Ω* or linear velocity *v*. The particle’s motion introduces a frequency shift across the scattered light. To analyze the total Doppler vector signal, we direct the scattered light through a linear polarizer oriented at an angle *θ* and detect it with a photodetector. The resulting time-related Doppler vector signal can thus be expressed as (see Supplementary Note 2),

2 $$\begin{array}{l}I(t)=8{|{A}_{0}(r)|}^{2}\left\{1+\mathop{\underbrace{\cos [({\ell }_{1}-{\ell }_{2}){\varphi }_{t}]}}\limits_{DS}+\mathop{\underbrace{\cos [2m{\varphi }_{t}+2({\varphi }_{0}+\sigma \theta )]}}\limits_{DPS}\right.\\ \left.+\mathop{\underbrace{\frac{1}{2}\left\{\cos [(2m-({\ell }_{1}-{\ell }_{2})){\varphi }_{t}+2({\varphi }_{0}+\sigma \theta )]+\,\cos [(2m+({\ell }_{1}-{\ell }_{2})){\varphi }_{t}+2({\varphi }_{0}+\sigma \theta )]\right\}}}\limits_{DPVSs}\right\}\end{array}$$

Therein, the time *t*-dependent phase *φ*_*t*_ = (*v/r*)*t* for translational motion (linear Doppler effect) or *φ*_*t*_ = *Ωt* for rotation (rotational Doppler effect), thus revealing their common physical origin^33^. From Eq. (2), the induced Doppler signal collapses into traditional linear/rotational DS if *m* = 0, *ℓ* ≠ 0, and degenerates into existing DPS when *m* ≠ 0, *ℓ* = 0. Only when both *m* ≠ 0 and *ℓ* ≠ 0, a novel type of DPVSs appear, thereby enabling a full characterization of existing Doppler effect. This universal formulation unifies traditional DS, established DPS, and the newly identified DPVSs under a single framework, each representing a specific case of the generalized expression, thus referred to as “GDS”. Crucially, while conventional DS depends solely on OAM (*ℓ*) and DPS arises exclusively from polarization (*m*), the DPVSs inherently couple both degrees of freedom, reflecting their simultaneous dependence on polarization and OAM.

The theoretical Doppler-shifted magnitudes for each regime within GDS can be derived from the phase differentials of their respective terms (Eq. 8, see Methods). Furthermore, Eq. (2) and (S13) (Supplementary Information) establish that the Doppler-shifted directionality of the GDS follows the sign of relative phase difference: $\varDelta \varphi \,=sign(\varDelta {f}_{DPS-th/DPVSs-th})\cdot 2(\varDelta {\varphi }_{0}+\sigma \varDelta \theta )$. Here, *sign*(·) denotes signum function, and *Δf*_*DPS-th/DPVSs-th*_ corresponds to Doppler-shifted magnitude of either DPS or DPVSs, respectively. This key relationship demonstrates that the red- or blue-shifted components of the GDS can be determined through measuring phase of either the initial polarization-angle offset *Δφ*_*0*_ or the linear polarizer-angle difference *Δθ* in two sequential measurements, hence eliminating the strict reliance on *Δθ* adjustments required in prior studies^23–25^.

A key feature of the Doppler shifts in Eq. (8) is that one DPVS component (DPVS1 or DPVS2) always exhibits a larger magnitude than both the DS and DPS (i.e., *Δf*_*DPVSs-th*_ >*Δf*_*DPS-th*_ and *Δf*_*DPVSs-th*_ > *Δf*_*DS-th*_). This amplified shift achieved via the combination of *m* and *ℓ*, reduces the relative measurement error under a given systematic error, thereby enhancing measurement accuracy. To quantify the enhanced efficacy of DPVSs on the measurement accuracy, we define two ratios of relative measurement errors for *Δf*_*DS-th*_ versus *Δf*_*DPVSs-th*_, *N*_*1*_, and *Δf*_*DPS-th*_ versus *Δf*_*DPVSs-th*_, *N*_*2*_,

3 $$\begin{array}{l}{N}_{1}=\frac{\delta {f}_{DS}}{\varDelta {f}_{DS-th}}/\frac{\delta {f}_{DPVSs}}{\varDelta {f}_{DPVSs-th}}={\kappa }_{1}(1+\frac{|m|}{\ell })\\ {N}_{2}=\frac{\delta {f}_{DPS}}{\varDelta {f}_{DPS-th}}/\frac{\delta {f}_{DPVSs}}{\varDelta {f}_{DPVSs-th}}={\kappa }_{2}(1+\frac{\ell }{|m|})\end{array}$$

Therein, *δf*_*DPS*_, *δf*_*DPVS1*_, *δf*_*DS*_, and *δf*_*DPVS2*_ are respectively absolute errors between measured and theoretical values for each Doppler shift, which are of comparable magnitude within a given measurement system, owing to consistent systematic noise. Consequently, the ratios of absolute error *κ₁* = *δf*_*DS*_ */ δf*_*DPVSs*_, *κ₂* = *δf*_*DPS*_ */ δf*_*DPVSs*_ are both approximately one. Here, *δf*_*DPVSs*_ = *δf*_*DPVS1*_ when *m* < 0, whereas *δf*_*DPVSs*_ = *δf*_*DPVS2*_ for *m* > 0. Furthermore, Eq. (3) reveals that DPVSs enhance the measurement accuracy by a factor of *κ₁*(1 + |*m*|/*ℓ*) over conventional DS sensing, and by *κ₂*(1 + *ℓ*/|*m*|) relative to current DPS approaches. Such enhancement can be determined by two factors: the absolute error ratio *κ* and the difference of |*m*| and *ℓ* (see Supplementary Notes3 and 4). Crucially, despite variations in accuracy-enhanced effect of DPVSs, there always exists that *N*_*1*_ > 0 and *N*_*2*_ > 0, regardless of |*m|* and *ℓ*, which thus represents an unprecedented enhancement in Doppler metrology accuracy. Overall, DPVSs provide a larger Doppler shift than conventional DP and existing DPS, which reduces relative measurement error and thus enhances Doppler-shifted measurement accuracy.

### Experimental setup

**Fig. 2:**
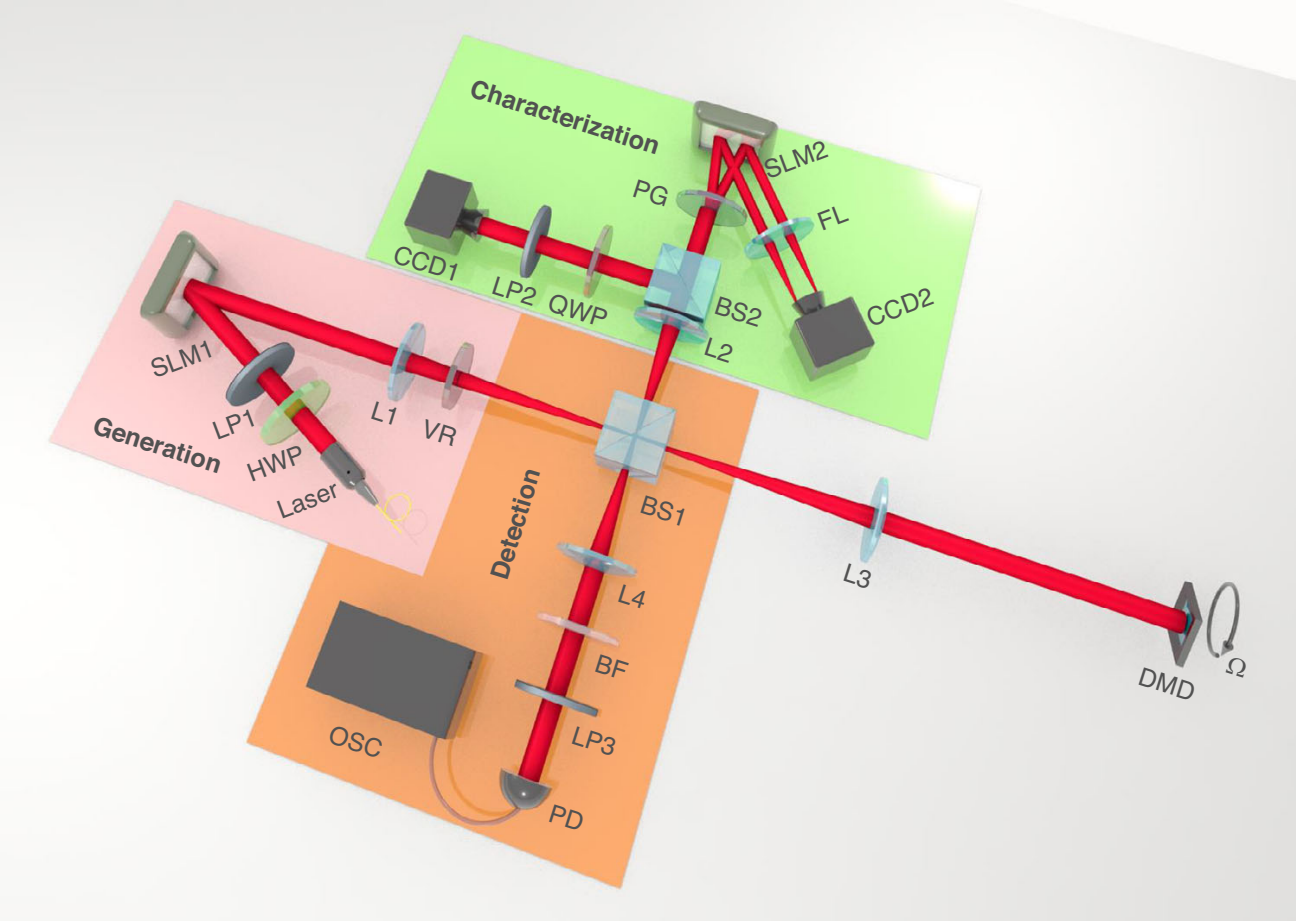
Experimental setup. HWP half-wave plate, LP1–LP3 linear polarizers, SLM1 and SLM2 spatial light modulators, VR vortex retarder, L1–L4 lenses, BS1–BS2 beam splitters, QWP quarter-wave plate, PG polarization grating, FL fourier lens, BF band-pass filter, PD photodetector, OSC oscilloscope, DMD digital micro-mirror device

To provide experimental validation of theoretical framework outlined above, we design and construct a robust experimental setup (Fig. 2) to thoroughly demonstrate the generalized Doppler effect and perform high-accuracy frequency shift measurements. A key element of the experiment is a programmable velocity emulator, implemented by a mirrored particle capable of executing both uniform and time-varying rotational motions (see “Methods”). Subsequently, the experimental workflow can thus be proceeded as follows. First, a novel structured light source of VPDVFs can be experimentally generated and the properties of polarization and OAM are respectively characterized prior to use. This tailored beam can then be directed onto the velocity emulator, where interaction with the moving surface imparted generalized Doppler shift to the incident light. And then, the backscattered signals are collected and pass through a polarization-resolved detection subsystem for the acquisition of GDSs. Finally, the GDSs corresponding to uniform motion are analyzed using the FFT operations, whereas those of time-varying motions are resolved via the short-time Fourier transform (STFT) and complementary signal-processing techniques (see Supplementary Note 5).

### Characterizations of VPDVFs

**Fig. 3:**
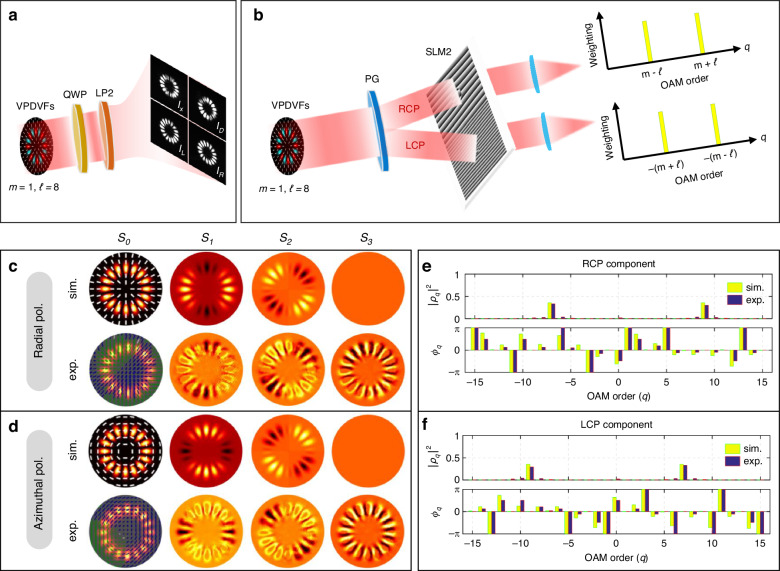
Polarization and OAM characterizations of generated VPDVFs. Schematic diagrams of (**a**) polarization characterization and (**b**) OAM characterization. *I*_*x*_, *I*_*D*_, *I*_*L*_ and *I*_*R*_ are horizonal, diagonal, left-handed and right-handed polarization intensities, respectively. Simulated and experimental Stokes parameter measurements for: (**c**) radially polarized (*m* = 1, *ℓ₁* = *-ℓ₂* = 8, *φ*_*0*_ = 0) and (**d**) azimuthally polarized (*m* = 1, *ℓ₁* = *-ℓ₂* = 8, *φ*_*0*_ = *π*/2) VPDVFs, respectively. Solid lines denote polarization states: white (linear), blue (left-handed circular), green (right-handed circular). OAM spectral analyses via (**e**) OAM power spectrum and (**f**) OAM phase spectrum for spin-OAM decoupled components of the radially/azimuthally polarized VPDVF with *m* = 1, *ℓ₁* = *-ℓ₂* = 8

We begin by characterizing optical properties of tailored VPDVFs through their polarization distributions and OAM spectra, including power and phase spectra, respectively (see Fig. 3a, b and “Methods”). The polarization properties are analyzed via simulated and experimental Stokes parameters (Fig. 3c, d and Supplementary Fig. S5a, b). The result shows that the *S*_*0*_ distributions correspond to the total intensity of the VPDVFs with superimposed polarization states. The *S*_*1*_ and *S*_*2*_ parameters exhibit mutually orthogonal patterns, which directly reflect the inherent orthogonality between the radial and azimuthal polarization components, respectively. This orthogonality arises because the polarization distributions of radial and azimuthal components are intrinsically perpendicular. This spatial polarization structure was further confirmed by linear polarizer measurements; that is, when set to horizontal and diagonal orientations, the filtered intensity patterns revealed the corresponding orthogonal local polarization states, a signature of classical nonseparability^34–36^. Meanwhile, the *S*_*3*_ parameter is negligible (*S*_*3*_ ≈ 0), indicating the absence of circular polarization components within the VPDVFs. Although the experimental results agree well with simulations, minor discrepancies are attributable to optical misalignments and imperfections in the optical elements.

We then characterize the OAM properties through simulated and experimental power and phase spectra in Fig. 3e, f and Supplementary Fig. S5c, d (see “Methods”). Unlike conventional phase-conjugated vortex fields, we find that the VPDVFs exhibit distinct OAM power spectral peaks. For right-handed circularly polarized (RCP) component, peaks emerge at the topological charges *q₂* = *m* + *ℓ* and *q₁* = *m* - *ℓ*; whereas left-handed circularly polarized (LCP) component shows peaks at *q₁* = -(*m - ℓ*) and *q₂* = -(*m* + *ℓ*), respectively. This unique behavior is attributed to the phase structures of the field components: exp[*i*(*ℓ₁+m*)*φ*)+exp(*i*(*ℓ₂+m*)*φ*] for RCP component, and exp[*i*(*ℓ₁-m*)*φ*)+exp(*i*(*ℓ₂-m*)*φ*) for LCP component in Eqs. (S2) and (S3), respectively. For instance, with the configured values *ℓ₁* = *-ℓ₂* = *ℓ* = 8 and *m* = 1 (Fig. 3e, f), the RCP’s spectrum shows peaks at 9 and −7, while LCP’s spectrum shows peaks at 7 and −9, as corroborated by simulations and measurements. These results confirm that the OAM spectra of the VPDVFs originate from spin-OAM coupling, distinct from that in scalar vortex or vectorially polarized fields^30–32^. Experimentally, we observe a slight spectral broadening around the predicted *q₁* and *q₂* peaks compared to simulations. Additionally, the measured OAM phase spectra, however, matched the simulations well at these spectral positions, with minor phase residuals attributable to noise.

### Experimental detection of GDSs and Doppler spectra

**Fig. 4:**
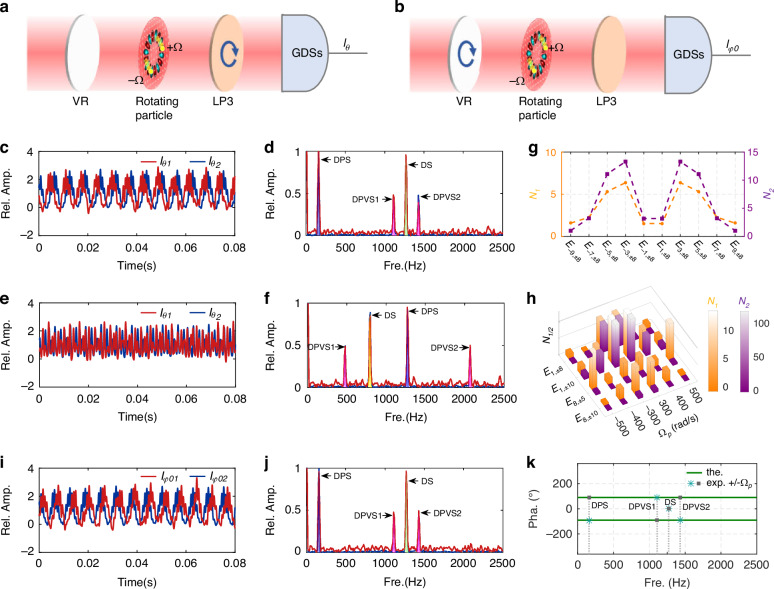
Experimentally measured GDSs and Doppler spectra. **a** Schematics of GDSs measurements by rotating LP3 with fixed VR, whereas (**b**) GDSs measurement by rotating VR with fixed LP3. **c**, **d** Measured GDSs through rotating LP3 (0° and 45°) for radially polarized VPDVF (*m* = 1, *ℓ₁* = *-ℓ₂* = 8, *φ*_*0*_ = 0), with the corresponding Doppler amplitude spectra exhibiting identical frequency shifts. **e**, **f** Measured GDSs through rotating LP3 (0° and 45°) for radially polarized VPDVF (*m* = 8, *ℓ₁* = *-ℓ₂* = 5, *φ*_*0*_ = 0), with corresponding Doppler amplitude spectra showing identical Doppler shifts. **g** Enhanced accuracy of DPVSs vs. DS, *N₁* = *κ₁*(1 + |*m*|/*ℓ*)), and DPVSs vs. DPS, *N₂* = *κ₂*(1 + *ℓ*/|*m*|). **h** Effect of preset rotational velocity vector and emitted light fields on enhancement accuracy. **i**, **j** Measured GDSs through rotating VR at initial polarization angles of 0° and 45° for radially polarized VPDVF (*m* = 1, *ℓ₁* = *-ℓ₂* = 8) with the fixed LP3 at angle of 0°, corresponding Doppler amplitude spectra possessing same Doppler shifts, and (**k**) relative phase difference. Theoretical phases: ±90° (green solid line); experimental values at reverse rotation directions (asterisks and squares)

Having characterized the emitted VPDVFs, we proceed to detect the generalized Doppler effect (see experimental details in Fig. 2 and “Methods”). As a representative example, a radially polarized VPDVF (*m* = 1, *φ*_*0*_ = 0, *ℓ₁* = *-ℓ₂* = 8) is launched onto a rotationally structured surface, which is programmed onto a digital micromirror device (DMD) with a preset angular velocity *Ω*_*p*_ = 500 rad/s. We experimentally validate GDSs and relative phase differences via a two-step procedure: first, VR is fixed at 0° while LP3 is rotated from *θ₁* = 0° to *θ₂*=45°; second, LP3 is fixed at 0° while VR is rotated from *φ₀₁* = 0° to *φ₀₂* = 45° (Fig. 4a, b). For the first step, the measured GDSs *I*_*θ₁*_ acquired with a linear polarizer (LP3) at 0°, clearly exhibits the temporal oscillations (Fig. 4c). FFT of this signal reveals four distinct peaks, which we identify as the established DS^16–18^ and DPS^23–25^, together with the two newly introduced DPVSs (DPVS1 and DPVS2) (Fig. 4d). The measured amplitudes of the DPVSs’ peaks are approximately half those of the DS and DPS, in quantitative agreement with the theoretical prediction from Eq. 2, thereby confirming the generalized Doppler effect. When the polarizer LP3 is rotated to 45°, the resulting signal *I*_*θ₂*_ exhibits phase delay/advance (Fig. 4c), while maintaining identical Doppler-shifted peak positions (Fig. 4d), consistent with previous reports^23,25^.

The generalized Doppler effect originates from the beating frequency after interference among the four OAM eigenmodes (*|*−7〉, *|*9〉, *|*−9〉, *|*7〉) identified within the OAM power spectrum (Fig. 3e, f). In this regard, we experimentally measure the Doppler shifts as Δ*f*_*DPS*_ = 160 Hz, Δ*f*_*DPVS1*_ = 1110 Hz, Δ*f*_*DS*_ = 1270 Hz, Δ*f*_*DPVS2*_ = 1430 Hz. These values show excellent agreement with the theoretical predictions from Eq. (8): Δ*f*_*DPS-th*_ = 159.15 Hz, Δ*f*_*DPVS1-th*_ = 1114.1 Hz, Δ*f*_*DS-th*_ = 1273.2 Hz, Δ*f*_*DPVS2-th*_ = 1432.4 Hz, with corresponding absolute errors of 0.85 Hz, 4.1 Hz, 3.2 Hz, and 2.4 Hz, respectively. The larger frequency shift of DPVS2 thereby enhances the measurement accuracy, and the enhanced ratios can be evaluated by a factor of *N*_*1*_ = 1.5 over the conventional DS and by *N*_*2*_ = 3.18 over the existing DPS, as follows by Eq. (3). Thus, the developed DPVSs universally provide superior accuracy compared to both traditional and established Doppler techniques.

To demonstrate the universality of the generalized Doppler effect, we prepared a VPDVF with a different combination of parameters (*m* = 8 and *ℓ*_*1*_ = *-ℓ*_*2*_ = 5), and measured their GDSs and Doppler spectra with the same experimental conditions (Fig. 4e, f). As predicted, the frequencies of the four Doppler peaks shift according to the altered parameters of the emitted field. The measured shifts are Δ*f*_*DPVS1*_ = 480 Hz, Δ*f*_*DS*_ = 800 Hz, Δ*f*_*DPS*_ = 1270 Hz, Δ*f*_*DPVS2*_ = 2070 Hz, with corresponding relative measurement errors of 0.53%, 0.51%, 0.25%, and 0.048%, respectively. The inverse correlation between the magnitude of the Doppler shift and its relative error is clearly evidenced, with the largest shift (DPVS2) yielding the highest accuracy. This result robustly confirms that the simultaneous optimization of *m* and *ℓ* provides a general and effective strategy for enhancing measurement accuracy.

Systematic evaluation confirms the consistent accuracy improvement of DPVSs over both DS and DPS (Fig. 4g). By characterizing VPDVFs across a range from *E*_*−9,±8*_ to *E*_*9,±8*_, we measure the corresponding enhancement factors. For instance, configurations *E*_3,±8_ and *E*_−3,±8_ yield a greater than 10-fold accuracy gain for DPVS over DPS. The relative superiority of DPVS depends on the interplay between |*m*| and *ℓ*: DPVS versus DPS dominates when |*m*| < *ℓ*, while DPVS versus DS prevails when |*m*| > *ℓ*, in full agreement with Eq. (3). Due to the non-negative frequency output of the FFT, configurations *E*_+*m,ℓ*_ and *E*_−*m,ℓ*_ yield identical enhancement factors. This systematic relationship provides a clear principle for selecting the optimal optical source for a given measurement. Furthermore, the enhanced accuracy of DPVSs proved robust across different rotational velocities and directions for multiple VPDVFs, including *E*_1,±8_, *E*_1,±10_, *E*_8,±5_, and *E*_8,±10_ (Fig. 4h). The enhancement factors *N*_1/2_ are independent of the rotation direction, as the absolute Doppler shift magnitudes remain unchanged. Notably, lower rotational velocities further improved accuracy by increasing the relative magnitude of the frequency shifts, thereby reducing the impact of measurement error. A striking demonstration of this principle is the case of *E*_1,±10_ results in a DPVS-versus-DPS accuracy enhancement exceeding two orders of magnitude (>100-fold), maximizing the Doppler shift separation as predicted by Eq. (3).

To probe directional sensitivity, we reverse the particle’s rotational velocity (±*Ω*_*p*_) and project the VPDVF of *E₁*, ± _*8*_ with initial polarization angles *φ₀₁* = 0° onto the rotating particle. The GDS measurements through LP3 at *θ₁* = 0° and *θ₂* = 45° reveal (Supplementary Fig. S6): (i) rotating the polarizer introduces a phase advance or delay in the GDS (Supplementary Fig. S6a, d); (ii) the Doppler amplitude spectra retain identical peak positions irrespective of the rotational direction (Supplementary Fig. S6b, e); and (iii) the Doppler phase spectra provide clear directional signatures, with the DPS and DPVS components exhibiting a phase difference of ~±*2Δθ*, while the DS component is directionally degenerate (Supplementary Fig. S6c, f), consistent with ref. ^23^. An equivalent directional discrimination is achieved by an alternative method by modulating VR. For the second step, we fix the polarizer LP3 at *θ₁* = 0° and instead modulated the initial polarization angle of the same VPDVF from *φ₀₁* = 0° to *φ₀₂* = 45° (Fig. 4b,i–k). The measured directional signatures are in good agreement with that of LP3 modulation, with the DPS and DPVS components exhibiting a phase difference of ~±*2Δφ₀*. Crucially, when |*m*| < *ℓ*, DPVS1 exhibits inverted directionality relative to DPS and DPVS2 (Fig. 4k), as governed by Eq. (2) and (S13). We note that experimental constraints limited *Δφ₀* to values other than 45° or 90°.

Overall, these results validate the generalized Doppler effect and demonstrate two distinct channels for directional sensing via either linear polarizer-angle difference (*Δθ*) or initial polarization-angle offset (*Δφ₀*). Within this framework, the DPVSs provide a remarkable accuracy enhancement exceeding 100-fold.

### Measurement of time-frequency spectra of GDS under time-varying motion

**Fig. 5:**
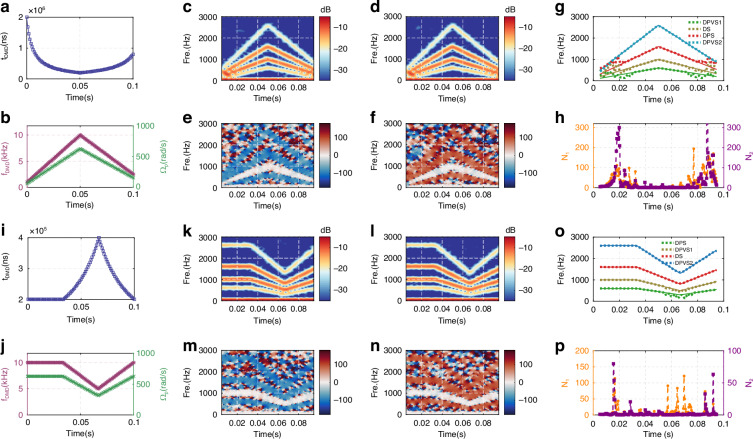
Experimental time-frequency analysis and performance evaluation for two distinct motion fashions. (**a, b, i, j**) The set DMD display duration *t*_*DMD*_ and corresponding refresh frequency *f*_*DMD*_ as well as preset rotational speed ±*Ω*_*p*_. (**c**, **d**, **k**, **l**) Time-frequency power spectra for ±*Ω*_*p*_, with *φ*_*01*_ = 0°, *φ*_*02*_ = 45°, *θ₁* = 0°, when emitting VPDVF with *m* = 8 and *ℓ₁*= -*ℓ₂* = 5. (**e**, **f**, **m**, **n**) Corresponding time-frequency phase spectra. (**g**, **o**) Theoretical vs. experimentally extracted time-varying Doppler shifts of GDSs. (**h, p**) Performance enhancement: DPVS vs. DPS (*N₁*) and DPVS vs. DS (*N₂*) ratios

To validate the performance of our approach under non-stationary conditions, we measured the generalized Doppler spectra from a particle with time-varying rotational speed. We programmed a DMD to simulate a motion profile of acceleration (0 ~ *T*/2) followed by deceleration (*T*/2 ~ *T*), with a total acquisition time *T* = 0.1 s over *N* = 100 frames per 2*π* period (see Fig. 5a and Experimental details in Methods). To avoid exceeding the DMD’s refresh limitation of 11.764 kHz, we dynamically adjusted *f*_*DMD*_ from 1 to 10 kHz (acceleration) and 10 to 2.5 kHz (deceleration), corresponding to *Ω*_*p*_ = 20π ~ 200π rad/s and 200π ~ 50π rad/s, respectively (Fig. 5b). Then, we illuminate the rotating structure with the VPDVF (*m* = 8, *ℓ₁* = −*ℓ₂* = 5) under opposite rotational directions at ±Ω, while modulating initial polarization angles (*φ₀₁* = 0° and *φ₀₂* = 45°) through VR (LP3 fixed at 0°). The STFT analysis of the retrieved signals reveals four distinct time-frequency trajectories in the Doppler power spectra for both positive (Fig. 5c) and negative rotation (Fig. 5d), confirming the persistence of the generalized Doppler effect under accelerated motion. The intensity distribution within these spectra allows clear identification of the DPVSs, DS, and DPS components. Furthermore, the simultaneous detection of all four Doppler signals within a single acquisition frame yields a fourfold increase in measurement efficiency compared to techniques relying on a single Doppler component.

Additionally, directional sensing is demonstrated by the relative phase difference spectra, *Δφ*, for +*Ω* (Fig. 5e) and −*Ω* (Fig. 5f), respectively. It can be found that DPS and DPVS components exhibit unambiguous directional signatures, with *Δφ* ≈ 90° for positive rotation and *Δφ* ≈ −90° for negative rotation. In contrast, the DS component remains directionally insensitive, showing *Δφ* ≈ 0° irrespective of the rotation direction. This establishes a method for directional sensing through emission-side polarization control via a VR, as an alternative to the detection-side polarizer rotation. We further evaluated the performance enhancement of DPVSs over DPS and DS. The spectral skeletons, extracted via a custom signal-processing algorithm (Fig. 5g and Supplementary Fig. S7), exhibit minor deviations at spectral convergence points due to the intrinsic resolution limit of the short-time Fourier transform. The experimental enhancement ratios, *N₁* (DPVS2 vs. DPS) and *N₂* (DPVS2 vs. DS), calculated from Eq. (3), systematically exceed unity across the measured range (Fig. 5h), confirming a consistent accuracy gain.

For the purpose of demonstrating the robustness of our developed generalized Doppler effect under complex, time-varying speeds, we programmed a three-stage motion profile from constant speed (0 ~ *T*/3, *Ω*_*p*_ = 200π rad/s), followed by deceleration (*T*/3 ~ 2 *T*/3, 200*π* → 100*π* rad/s), and finally acceleration (2 *T*/3 ~ *T*, 100*π* → 200*π* rad/s), respectively (Fig. 5i, j). The total acquisition time and the illuminating VPDVF remain unchanged. Correspondingly, *f*_*DMD*_ after setting *t*_*DMD*_ maintains 10 kHz (0 ~ *T*/3), 10 kHz→5 kHz (*T*/3 ~ 2 *T*/3) and 5 kHz→10 kHz (2 *T*/3 ~ *T*), respectively. The resulting time-frequency spectra retain all characteristic features (Fig. 5k–n), and the extracted enhancement factors remain positive with *N₁* > 0 and *N₂* > 0 throughout the entire motion sequence (Fig. 5o, p), conclusively validating the accuracy-enhanced efficacy of DPVSs in dynamic sensing scenarios.

## Discussion

In conclusion, we have established a universal framework of the generalized Doppler effect and demonstrated a pathway to significantly enhanced measurement accuracy, based upon structured light-particle interactions and Doppler-shifted amplification. To achieve these, we first generated a novel class of VPDVFs leveraging the spin-orbit coupling principle. Digital modal decomposition revealed a unique OAM spectrum within these fields, characterized by OAM orders *q₂* = *m* + *ℓ* and *q₁* = *m* − *ℓ* for the RCP components, and *q₁* = −(*m* − *ℓ*) and *q₂* = −(*m* + *ℓ*) for the LCP components. The interaction of these VPDVFs with moving particles yields a versatile GDS, from which we simultaneously extract four distinct Doppler spectra under both constant and time-varying motion, including the new member of the Doppler “family”, DPVSs. Furthermore, we demonstrated that the initial polarization angle difference (*Δφ*_*0*_) can be served as an additional parameter for discriminating red/blue shifts in GDS, complementing the conventional linear polarization angle difference (Δ*θ*). This enhanced directional discrimination strategy has the potential to distinguish positive and negative refractive index materials^37^. Most critically, the developed DPVSs achieve an unprecedented enhancement in measurement accuracy, delivering a *κ₁*(1 + |*m*|/*ℓ*)-fold improvement over traditional DS metrology and *κ₂*(1 + *ℓ*/|*m*|) times enhancement compared to current DPS metrology. While the upper limit of GDS metrology is primarily constrained by the combined polarization and OAM orders achievable via SLM and VR for emission field manipulation, the underlying principle of generalized Doppler metrology can be extensible to arbitrary structured light fields. We anticipate that these findings will open new avenues for high-accuracy Doppler metrology across diverse applications, including fluid vorticity measurement^38^, hemotachometry^3^, biomedical diagnostics^39^, meteorological sensing^5,40–43^, and celestial body detection^44,45^, etc.

Regarding the application of the generalized Doppler effect in the future, our findings particularly illuminate the promise of the newly identified vector-vortex Doppler components. Its principal advantage lies in a substantially amplified frequency shift enabled by spin-orbit coupling, compared to conventional rotational or vectorial Doppler effects. However, translating this advantage into practical sensing scenarios introduces a key challenge. That is, when the target is a diffuse reflector, the backscattered light field contains multiple spatial modes, where the beating frequency between these modes broadens generalized Doppler spectrum while the weakened return signals degrade the signal-to-noise ratio. To overcome this limitation, mode-selective filter offers a targeted solution. By employing modal filters designed to match the known spatial structure of the emitted vector-vortex field, the specific vector-vortex Doppler signal peak corresponding to the desired mode can be isolated within the demodulated signal. This strategy suppresses competing spectral components, thereby enabling accurate frequency-shift extraction and enabling higher-accuracy velocity measurements even under non-ideal scattering conditions.

## Materials and methods

### Characterizations of polarization and OAM

Here, we characterize the polarization states of the VPDVFs through simulation and measurement of four Stokes parameters^46–48^:

4 $$\begin{array}{lll}{S}_{0}={I}_{R}+{I}_{L}\\ {S}_{1}=2{I}_{x}-{S}_{0}\\ {S}_{2}=2{I}_{D}-{S}_{0}\\ {S}_{3}={I}_{R}-{I}_{L}\end{array}$$

Here, *I*_*R*_, *I*_*L*_, *I*_*x*_ and *I*_*D*_ denote the intensities of RCP, LCP, horizontal and diagonal polarization components, respectively. In simulation, *I*_*R*_ and *I*_*L*_ can be calculated by Eqs. (S1-S3) (Supplementary Note 1). *I*_*x*_ = |*E*_*x*_|² calculated by Eq. (S4), and *I*_*D*_ = |*E*_*R*_ + *iE*_*L*_|². In the experiment, Stokes parameters were acquired using a quarter-wave plate (QWP) and linear polarizer in sequence. *I*_*x*_ and *I*_*D*_ are measured by projecting the generated VPDVF through a linear polarizer at 0° and 45°; *I*_*R*_ and *I*_*L*_ are obtained with QWP at 45° and 135°, followed by linear polarizer at 90°, respectively. Eventually, the polarization distributions can be reconstructed from experimentally measured four Stokes parameters (Fig. 3a, b).

To characterize the OAM property of VPDVFs, we calculate and measure OAM power and phase spectra based upon the modal decomposition principle, respectively. In simulations, we decompose VPDVFs into RCP/LCP components *e*_*±σ*_, and project them onto a set of orthogonal basis vectors. Here, we select *LG*_*p=0,q*_ modes with completeness and orthogonality as the OAM basis vectors to filter out their relative weightings within VPDVFs. The calculated OAM power spectrum can be expressed as:

5 $$\begin{array}{l}{|{C}_{q}|}^{2}={|\langle {\varPhi }_{q}^{\ast }|{e}_{+/-\sigma }\rangle |}^{2}\,=\,\frac{1}{2}{|\langle {\varPhi }_{q}^{\ast }|{e}_{x}\rangle |}^{2}\\ =\,\frac{1}{2}{|\iint {\varPhi }_{q}^{\ast }(r,\varphi ){e}_{x}(r,\varphi )rdrd\varphi |}^{2}\\ =\frac{\sqrt{2}}{4}{|\iint {e}^{-iq\varphi }{A}_{0}(r)({e}^{i\ell \varphi }+{e}^{-i\ell \varphi }){e}^{i\sigma (m\varphi +{\varphi }_{0})}rdrd\varphi |}^{2}\\ =\frac{\sqrt{2}}{4}{I}_{0}(r){|{\int }_{0}^{2\pi }{e}^{i\sigma {\varphi }_{0}}[{e}^{i(-q+\sigma m+\ell )\varphi }+{e}^{i(-q+\sigma m-\ell )\varphi }]d\varphi |}^{2}\\ =\sqrt{2}{\pi }^{2}{I}_{0}(r){|{e}^{i\sigma {\varphi }_{0}}({\delta }_{q,\sigma m+\ell }+{\delta }_{q,\sigma m-\ell })|}^{2}\\ =\left\{\begin{array}{l}\sqrt{2}{\pi }^{2}{I}_{0}(r){({\delta }_{q,m+\ell }+{\delta }_{q,m-\ell })}^{2},\sigma =1\\ \sqrt{2}{\pi }^{2}{I}_{0}(r){[{\delta }_{q,-(m-\ell )}+{\delta }_{q,-(m+\ell )}]}^{2},\sigma =-1\end{array}\right.\end{array}$$

Therein, only *e*_*x*_ component within Eq. (S3) can be filtered out within each divided RCP/LCP component during projection measurement via SLM due to its operation principle, thus reducing total detected power to 50% of circular polarizations. *Φ*_*q*_ = *LG*_*p=0,q*_ = exp(*-iqφ*) representing the basis vectors. *δ*_*a,b*_ is the Kronecker delta function, where only if *a* = *b*, *δ*_*a,b*_ = 1, otherwise *δ*_*a,b*_ = 0. Equation (5) confirms two eigenmodes per polarization component: *m* + *ℓ* and *m* − *ℓ*, for RCP component and −(*m* − *ℓ*) and −(*m* + *ℓ*), for LCP component.

Aside from OAM power spectrum, we also consider the phase spectrum (i.e., intramodal phase spectrum) of VPDVFs. Theoretically, the OAM phase spectrum can be given as follows:

6 $$\begin{array}{l}{\phi }_{q}^{sim}=\frac{\Re ({C}_{q})}{\Im ({C}_{q})}=\frac{2\pi {I}_{0}(r)cos(\sigma {\varphi }_{0})({\delta }_{q,\sigma m+\ell }+{\delta }_{q,\sigma m-\ell })}{2\pi {I}_{0}(r)sin(\sigma {\varphi }_{0})({\delta }_{q,\sigma m+\ell }+{\delta }_{q,\sigma m-\ell })}\\ \,=\left\{\begin{array}{l}\cot ({\varphi }_{0}),\sigma =1\\ \cot (-{\varphi }_{0}),\sigma =-1\end{array}\right.\end{array}$$

with ℜ and ℑ denote real and imaginary parts. From Eq. (6), we can see that the intramodal phases depend solely upon initial polarization angle *φ*_*0*_. That is, regardless of emitting any polarized VPDVF (*φ*_*0*_ = 0 or *φ*_*0*_ = π/2), their OAM phase spectra are always null for two orthogonal circularly polarized components.

Experimentally, we validate the predicted OAM phase spectrum using multiplexed computer-generated hologram. The power spectra are acquired by encoding OAM orders *q* ∈ [−15, 15] on SLM. Then, we measure on-axis intensity for each OAM basis vector, synchronously. To measure OAM phase spectrum, we require four conjugate modes *E*_*sin*_, *E*_*cos*_, *Φ₀* and *Φ*_*q*_ per mode *q*. Among them, the interference modes *E*_*sin*_ = *Φ*_*q*_ + *Φ₀* and *E*_*cos*_ = *Φ*_*q*_ + *iΦ₀*, with *q*-order eigenmode *Φ*_*q*_ and referred Gaussian mode *Φ₀*, respectively. And thus, the OAM eigenmode phase that consists of OAM phase spectrum can be extracted by:

7 $${\phi }_{q}^{exp}=\,arctan\left(\frac{2{I}_{sin}-{I}_{0}-{I}_{q}}{2{I}_{cos}-{I}_{0}-{I}_{q}}\right)$$

where *I*_*sin*_, *I*_*cos*_, *I*_*0*_, and *I*_*q*_ denote four measured on-axis intensities.

### Experimental details

The proof-of-concept experimental setup consists of four functional modules (Fig. 2): generation, characterization, detection and velocity emulator. (i) A 1550 nm single-longitudinal-mode laser first passes through a half-wave plate (HWP) and a linear polarizer (LP1) set to 0° to control output power and polarization state. The beam then illuminates a phase-only reflective SLM1, on which a preloaded, computer-generated hologram (CGH) is written using a complex-amplitude-modulation technique. This CGH converts the incident Gaussian mode into phase-conjugated vortex modes. Subsequently, the modulated beam traverses a liquid-crystal vortex retarder (VR), yielding a vector-polarization-distributed vortex field (VPDVF) with independently tunable polarization order *m* and OAM order *ℓ*. (ii) To characterize the polarization and OAM properties, the generated VPDVF is split by a first beam splitter (BS1) and directed into two parallel arms after recombination at BS2. In the reflected arm of BS2, polarization analysis is performed via a second linear polarizer (LP2), a quarter-wave plate (QWP), and CCD1, following standard Stokes polarimetry (Eq. (4)). In the transmitted arm, the OAM spectrum is measured through eigenmode decomposition (Eqs. (5) and (7)): a polarization grating (PG) separates the VPDVF into left- and right-circular polarization components, which are then projected onto separate regions of a second reflective SLM (SLM2). A Fourier lens (FL) and CCD2 record the corresponding intensity patterns to extract both power and phase distributions of each OAM mode. (ⅲ) After these, to verify the generalized Doppler effect experimentally, the VPDVF is imaged through a 4 f system comprising two plano-convex lenses (L1, L3; *f₁* = *f₂* = 200 mm) onto a rotation emulator. The back-scattered or reflected light is then collected by a second 4 f arrangement (L3–L4; *f₃* = 100 mm) and directed to a photodetector (PD) and an oscilloscope (OSC). A 4 nm-bandwidth band-pass filter (BF) suppresses ambient background, while an additional linear polarizer (LP3) enables selective analysis of the polarization-resolved Doppler signals. (ⅳ) A digital micro-mirror device (DMD, 1280 × 800 pixels, 10.8 µm pixel pitch) serves as the rotating target. Twenty adjacent micromirrors form a reflective “particle” of radius 216 µm, which follows a circular trajectory of 3 mm for VPDVF with *m* = 1, *ℓ₁* = −*ℓ*₂ = 8 or 2.45 mm for *m* = 8, *ℓ₁* = −*ℓ₂* = 5. Rotation is emulated by sequentially displaying *N* binary images over a 2π cycle, yielding the angular frequency: *Ω* = 2*π*/[(1/*f*_*ref*_)*N*] = 2*πf*_*ref*_*/N*. Here, *f*_*ref*_ = 11.764 kHz is the maximum DMD refresh ratio. In practice, *N* = 149 binary frames produce *Ω*=500 rad/s; reversing the image order inverts the rotation direction. For uniformly accelerated or decelerated motion, the total frame count *N*′ remains fixed over 2*π* while the instantaneous speed varies linearly from *Ω*_*0*_ to *Ω*_*e*_ over time *T*′. Such that the acceleration/deceleration is given by *var* = (*Ω*_*e*_ − *Ω₀*)*/T’*. Consequently, the rotational speed at each step is *Ω*_*a/d*_ = *Ω₀* + *varT’*. And thus, the corresponding DMD refresh frequency is *f*_*DMD*_ = *Ω*_*a/d*_ *N*′/(2*π*), resulting in DMD display duration of preset each picture *t*_*DMD*_ = *2/f*_*DMD*_. Since 2 × 10^9 ^ns corresponds to 1 Hz within our DMD controller, doubling the frame duration accommodates the desired variable rotation profile.

### Theoretical analysis of Doppler shifts

According to Eq. (2), when the phase evolves as *φ*_*t*_ = *Ωt*, the theoretical Doppler shifts for the DPS, the two DPVSs, and the conventional DS are given by: Δ*f*_*DPS-th*_ = |2 *m*|*Ω*/2*π*, Δ*f*_*DPVS1-th*_ = |2 *m -* (*ℓ₁* - *ℓ₂*)|*Ω*/2*π*, Δ*f*_*DS-th*_ = |*ℓ₁* − *ℓ₂*|*Ω* / 2*π* and Δ*f*_*DPVS2-th*_ = |2 *m* + (*ℓ₁* − *ℓ₂*)|*Ω* / 2*π*, respectively. In order to maximize the frequency shift, we set *ℓ₁* = *-ℓ₂* = *ℓ* (*ℓ* > 0) to improve the measurement accuracy due to the double frequency shift^14^. Thus, the Doppler shifts of GDS can be rewritten as follows:

8 $$\begin{array}{lll}\varDelta {f}_{\mathrm{DPS}-th}&=&|\frac{m\varOmega }{\pi }|\\ \varDelta {f}_{\mathrm{DPVS}1-th}&=&|\frac{(m-\ell )\varOmega }{\pi }|\\ \varDelta {f}_{\mathrm{DS}-th}&=&|\frac{\ell \varOmega }{\pi }|\\ \varDelta {f}_{\mathrm{DPVS}2-th}&=&|\frac{(m+\ell )\varOmega }{\pi }|\end{array}$$

From Eq. (8), when *m* > 0, Δ*f*_*DPVS2-th*_ exceeds both Δ*f*_*DS-th*_ and Δ*f*_*DPS-th*_, while if *m* < *0*, Δ*f*_*DPVS1-th*_ dominates. This demonstrates that our DPVS schemes inherently yield larger frequency shifts than either traditional DS or existing DPS, and thus finer resolution.

## Supplementary information

Supplementary information for Generalized Doppler Effect for High-accuracy Frequency Shift Measurement

## Data Availability

The authors declare that all relevant data are available in the paper and Supplementary Information, or from the corresponding author on request.
