# Supplementary material for: Generalized Doppler effect for high-accuracy frequency shift measurement
Source: Light Sci Appl. 2026 Apr 13;15:197. doi: 10.1038/s41377-026-02259-9 (PMC13076828; doi:10.1038/s41377-026-02259-9)
Supplement: Supplementary file 1 — Supplementary information for Generalized Doppler Effect for High-accuracy Frequency Shift Measurement [file 41377_2026_2259_MOESM1_ESM.docx]

**Supplementary Information for**

****Generalized Doppler Effect for High-accuracy Frequency Shift Measurement****

Yanxiang Zhang^1,2,∗^, Dexin Ba^1,2^, YangYang^2,∗^ and Yongkang Dong^1,2,∗^

*^1^National Key Laboratory of Laser Spatial Information, Harbin Institute of Technology, Harbin 150001, China*

*^2^Zhengzhou Research Institute, Harbin Institute of Technology, Zhengzhou 450007, China*

This PDF includes:

Supplementary Notes 1~5;

Supplementary Figures S1~S7.

**Supplementary Note 1: Theoretical generation of vectorially polarized dual-vortex fields (VPDVFs)**

In the main text (Fig. 1c), we launch a vectorially polarized dual-vortex field (VPDVF) onto a homogeneous isotropous particle surface and observe its induced generalized Doppler effect. In the cylindrical coordinates (*r, φ, z* = 0), the incident VPDVF can be written as,

 (S1)

where *A_0_* is the uniform amplitude. *E_DV_* denotes dual-vortex light fields, which can be manifested as:

 (S2)

with *ℓ₁*, *ℓ₂* being the orbital angular momentum (OAM) orders. *P* stands for the vectorial polarization component, which can be formulized as:

 (S3)

Here, *m* is the polarization order, *φ₀* is the initial polarization angle. *e_L_* and *e_R_* as well as *e_x_* and *e_y_* are respectively two pairs of orthogonal unit vectors. *σ* = +1 or -1 represents right-hand circular polarization or left-hand circular polarization. Substituting Eqs. S2 and Eq. S3 into Eq. S1, one can obtain the created VPDVFs:

 (S4)

From Eq. S4, we can see that when *m* ≠ 0 and *φ₀*=0, the generated light fields reduce to a radially polarized dual-vortex field, whereas if *m* ≠ 0 and *φ₀*=*π*/2, it becomes an azimuthally polarized dual-vortex light field. When *m*=0, Eq. S4 can be simplified as:

 (S5)

Here, *e_x_* is the unit vector along *x*-direction. In that time, the created VPDVFs degrade into scalar light fields. In addition, when *ℓ₁=ℓ₂=ℓ*, Eq. S4 can be simplified as:

 (S6)

That is, the developed VPDVFs degenerates further into vectorially polarized vortex fields. When *ℓ₁=-ℓ₂=ℓ*, Eq. S4 recasts as vectorially polarized phase-conjugated vortex fields:

 (S7)


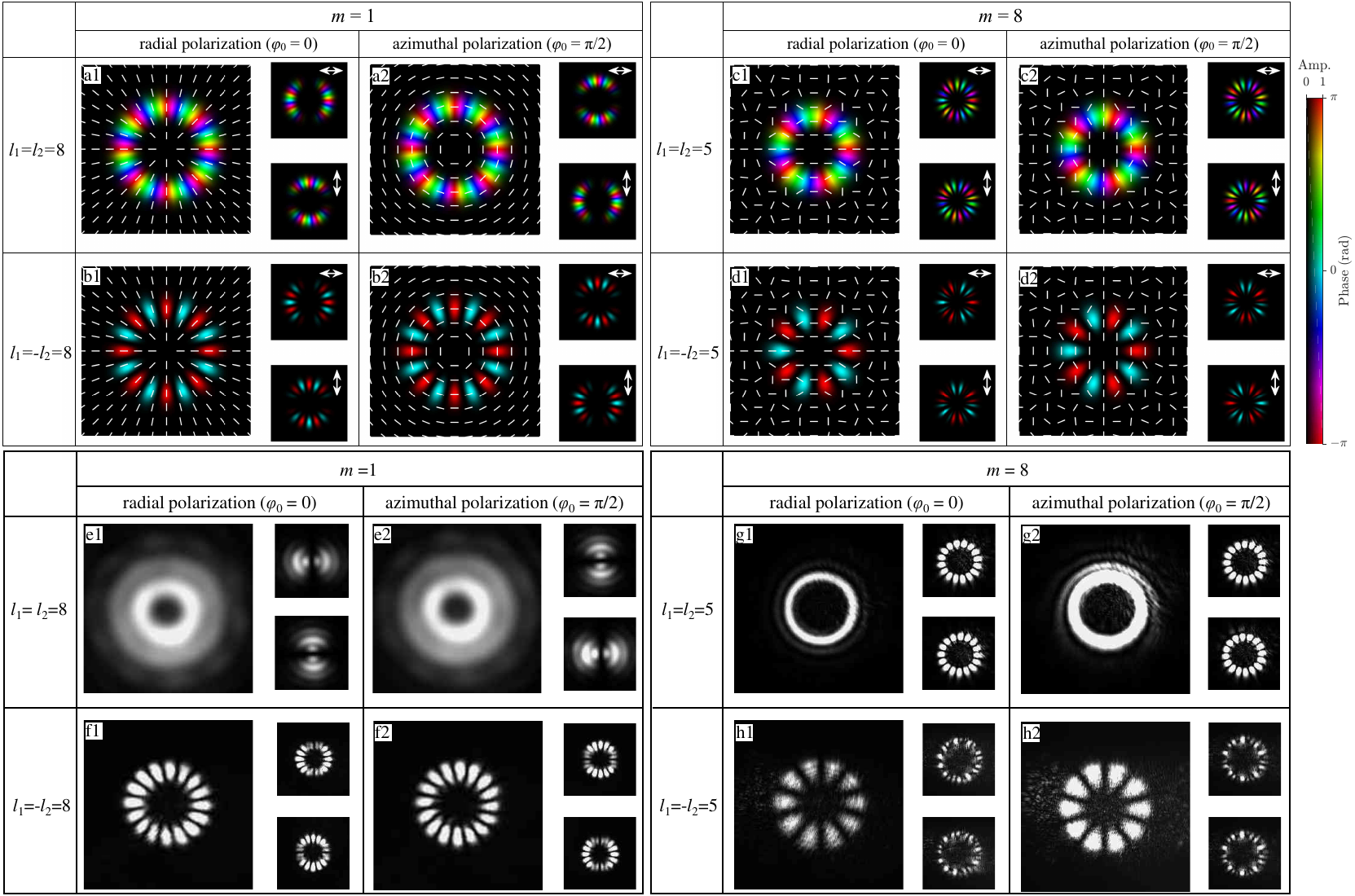


**Supplementary Fig. S1 Manipulation and control of VPDVFs.** (**a1,a2;e1,e2**) The simulated and experimental fields possess OAM order*ℓ_1_​=ℓ_2_​*=8 with radially polarized (*m*=1,*φ_0_*=0) and azimuthally polarized (*m*=1,*φ_0_*=*π*/2) states. (**b1,b2;f1,f2**) The simulated and experimental fields with phase-conjugated OAM order *ℓ_1_​=−ℓ_2​_*=8 and with radially polarized (*m*=1,*φ_0_*=0) and azimuthally polarized (*m*=1,*φ_0_*=*π*/2) states. (**c1,c2;g1,g2**) The simulated and experimental fields with OAM order *ℓ_1_​=ℓ_2_​*=5 and with radially polarized (*m*=8,*φ_0_*=0) and azimuthally polarized (*m*=8,*φ_0_*=*π*/2) states. (**d1,d2;h1,h2**) The simulated and experimental fields with phase-conjugated OAM order *ℓ_1_​*=−*ℓ_2_​*=5 and with radially polarized (*m*=8,*φ0*=0) and azimuthally polarized (*m*=8,*φ_0_*=*π*/2) states. In each panel, the inset at top-right shows the *x*-component and the inset at bottom-right shows the *y*-component, obtained by inserting horizontal and vertical linear analyzers (white solid lines denote the direction of a polarization analyzer). Here, the simulated parameters are selected as follows: laser central wavelength *λ*=1550 nm, beam waist radius *w*_0_​=1 mm.

To clearly visualize the created VPDVFs predicted by Eq. S4, we perform numerical simulations of the total fields *I*, its orthogonal component fields *I_x_* and *I_y_*, and the corresponding phase distributions, while simultaneously resolving the spatially varying polarization via orthogonal polarization analyzers. Here, *I*=*I_x_*+*I_y_*, *I_x_*=|*E_x_*|^2^ and *I_y_*=|*E_y_*|^2^. The phase distributions are given by *arctan*(ℑ(*E*)/ℜ(*E*)), where ℑ and ℜ respectively denote the imaginary and real parts of complex light fields. The simulated and experimental results are shown in Fig. S1. We can observe the total fields and two component fields concerning *E_ℓ_*​ and *E_±ℓ_*​ (here *ℓ*=8). It can be seen that when *ℓ_1_=ℓ_2_*=8 and *ℓ_1_=ℓ_2_*=5 (Figs.S1 a1–a2 and c1–c2; e1–e2 and g1–g2), their intensity distributions always show hollow rings and the phase varies from 0 to 2*π*. The directions of polarization analyzer show the effect of intensity-dimensional filter on the corresponding polarization directions, which can be referred as "nonseparability" or "classical entanglement" [1-3]. For comparison, when *ℓ_1_=−ℓ_2_*=8 and *ℓ_1_=−ℓ_2_*=5 (Fig. (Figs. S1 b1–b2 and d1–d2; f1–f2 and h1–h2), their intensity distributions always show petal shapes and the phase varies from 0 to *π*. The intensity corresponding superposed polarization directions also can be filtered via a polarization analyzer, which are *x*- and *y*-component fields of total fields, respectively.

**Supplementary Note 2: Generalized Doppler effect of VPDVFs**
When we guide such generated VPDVFs onto a moving object surface, such motion shifts the frequency of incident light and hence results in a generalized Doppler effect within the scattered light. To detect this effect, we further guide the scattered generalized Doppler signal to pass through a polarizer with the polarization angle of θ for the discrimination of motion directions. As a consequence, the final light intensity of the scattered light fields can be collected by the beating frequency effect. This process can be formulized by the Jones matrix representation as the following description:

 (S8)

In Eq. (S8), φ*_t_* is a time-modulated phase resulting from the motion of an object, which can be given as φ*_t_* =(*v*/*r*)*t* for a translated particle with the translational velocity of *v*, or φ*_t_*=*Ωt* for a rotational particle with the rotational velocity of Ω. Here, *k* is the wave vector, and α is the angle between the Poynting vector and the light axis. Furthermore, the second term of Eq. (S8) for phase-conjugated vortex light fields can be further simplified as the following:

 (S9)

and the last term of Eq. (S8) can be given as:

 (S10)

As a result, Eq. (S8) can be rewritten as:

(S11)

In Eq. (S11), there exist four main terms, including traditional linear/rotational Doppler signal (DS), Doppler polarization signal (DPS), and our new-developed Doppler polarization-vortex signal (DPVS1 and DPVS2). This outcome covers all existing Doppler effects, and hence we call it the Generalized Doppler Effect. Since the term *e^i^*^(ℓ1+ℓ2)φ^*^t^* is the high-frequency component and thus the photodetector in real detection scenarios fails to respond to this component, this factor can be ignored in detected GDSs.


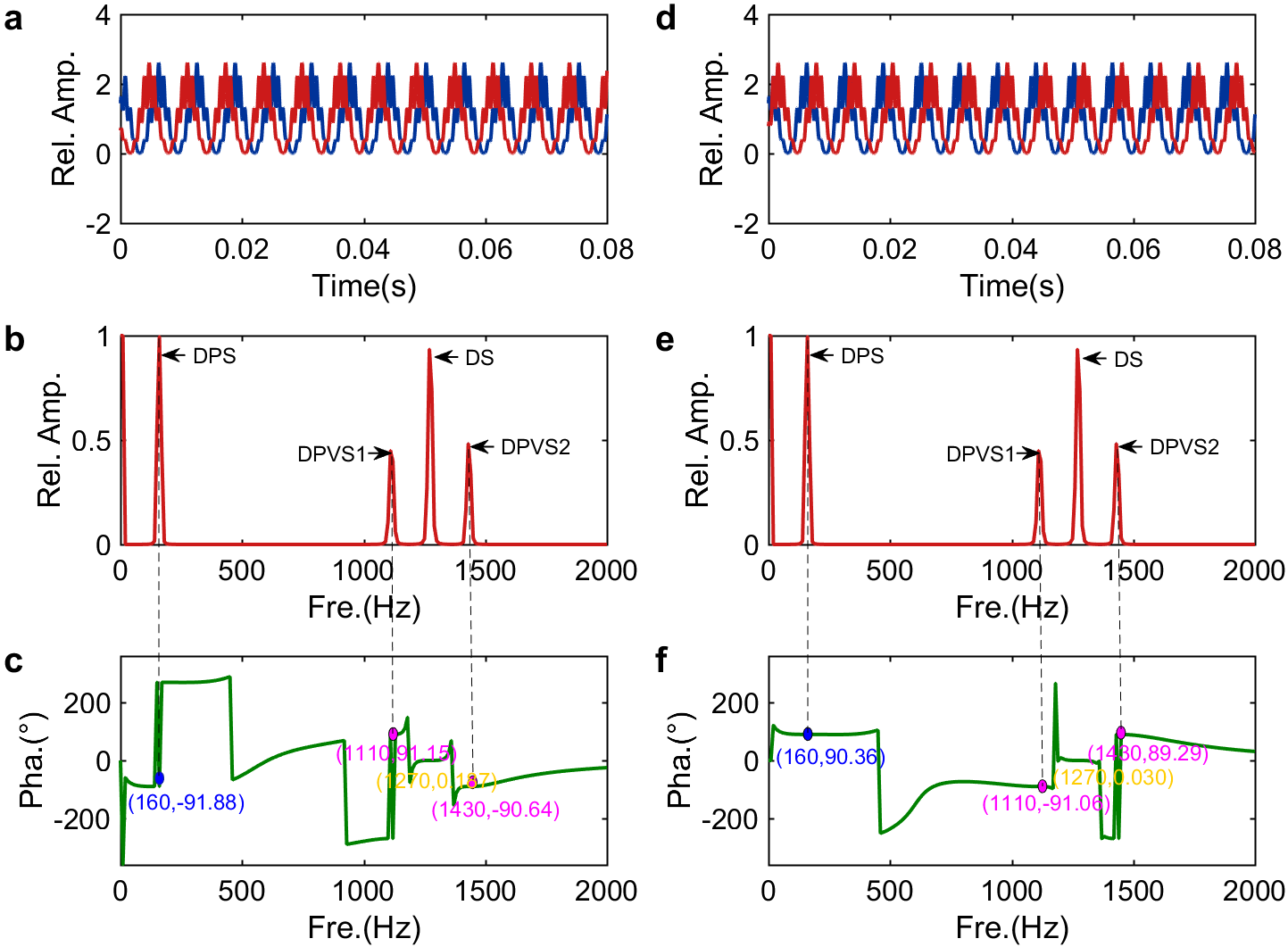


**Supplementary Fig. S2 Numerically simulated generated Doppler effect.** (a)-(c) generalized Doppler signals, generalized Doppler spectra and relative phase difference when rotational velocity is simulated at +500 rad/s, the initial polarization angles at 0° and 45° for radially polarized VPDVF with m=1, ℓ₁=-ℓ₂=8, respectively. (a)-(c) generalized Doppler signals, generalized Doppler spectra and relative phase difference when rotational velocity is simulated at -500 rad/s, the initial polarization angles at 0° and 45° for radially polarized VPDVF with m=1, ℓ₁=-ℓ₂=8, respectively.

**Supplementary Note 3: Doppler-shifted vector of DPVSs**
One can see from Eq. (S11) that the new DPVSs include two terms, where the former's phase is [2*m*−(*ℓ_1_−ℓ_2_*)]φ*_t_* and the latter's phase is [2*m*+(*ℓ_1_− ℓ_2_*)]φ*_t_*. This means that the frequencies of the two DPVSs shift (*ℓ_1_−ℓ_2_*)φ*_t_* symmetrically around 2*m* (corresponding to DPS) on the frequency axis. Their initial phase 2(φ_0_+σθ) can be used to characterize red/blue shift, respectively. As shown in Fig. S3a, assuming that the normally illuminated particle rotates within a 2-dimensional plane, i.e., φ*_t_*=*Ωt*, the frequency shifts of DPVSs can be given as the differential of phase with respect to time, respectively:

 (S12)

In Fig. S3b, the directions of these two Doppler shifts can be determined by the initial phase difference Δφ, after two-step Fourier phase spectrum measurements with initial polarization angles φ_01_ and φ_02_ (corresponding to VR within Fig. S2), or linear polarization angles θ*_1_* and θ_2_ (corresponding to LP3 within Fig. S1), respectively:

 (S13)

Therein, sign(·) represents the symbolic function. Δφ*_0_*=φ*_01_*− φ*_02_*, and Δθ = θ*_1_*− θ*_2_*. If we fix φ*_01_*= φ*_02_*=0 (i.e., radially polarized VPDVFs), Δφ is mainly determined by the difference between two linear polarization angles Δθ, which is consistent with Ref. [4-6]. In contrast, when we fix θ*_1_*= θ*_2_* = 0 (i.e., maintaining the horizontal polarization of LP3), Δφ can also be acquired by rotating VR two times with the difference of initial polarization angles Δφ*_0_*. This is a new finding in this paper. Note that none of Δφ*_0_* or Δθ can be set as 0 or π/2, otherwise the directions cannot be distinguished due to the signals' periodicity. Additionally, we can observe that the amplitudes of the two DPVSs are half of those of DS or DPS, whose distribution law can be used to identify themselves.


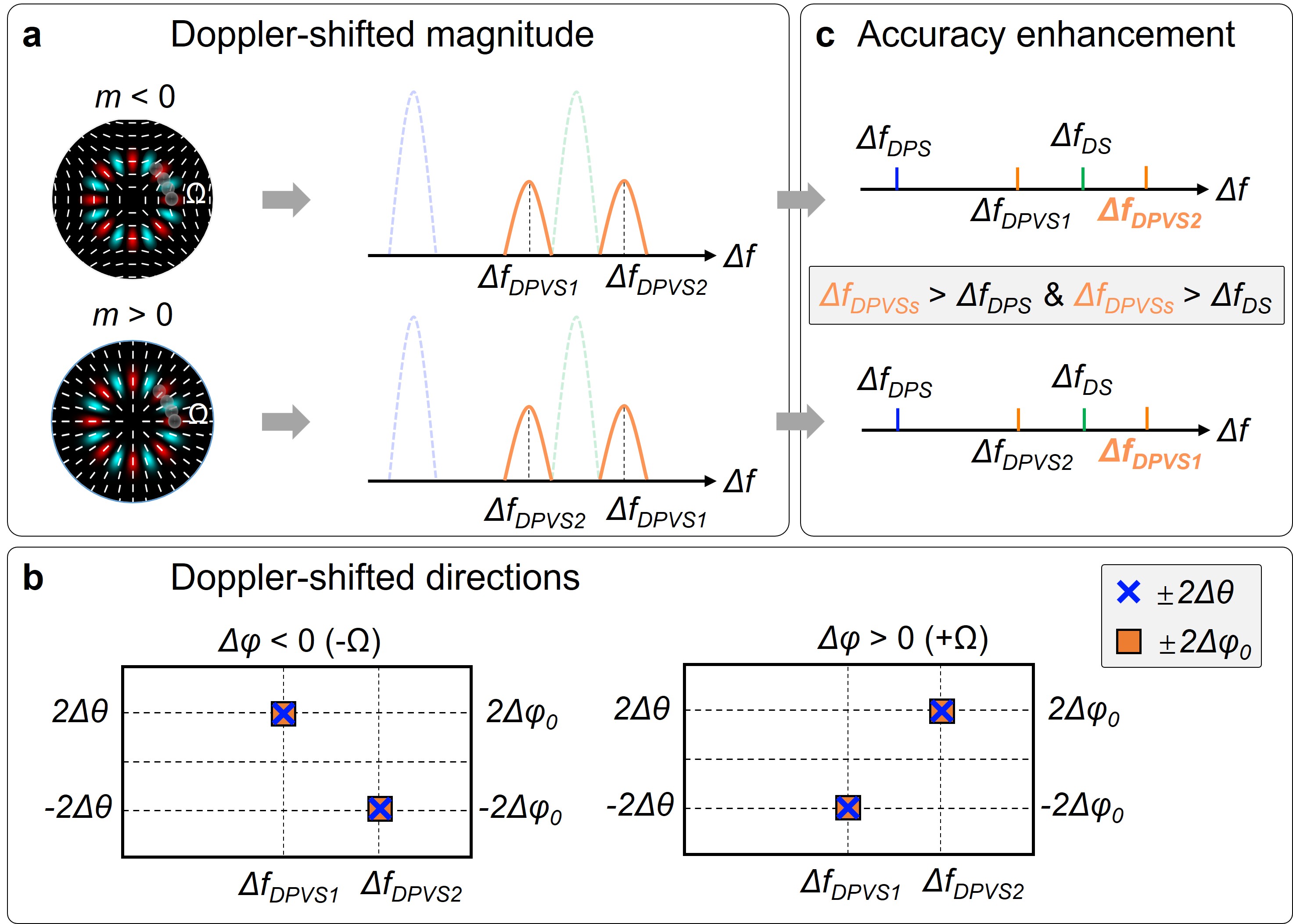


**Supplementary Fig. S3 Doppler-shifted magnitude and directions of DPVSs and its accuracy-enhanced measurement.** (**a**) Doppler-shifted magnitude *f_DPVSs_* of DPVSs, when the polarization order *m* > 0 and *m* < 0. (**b**) Discrimination of Doppler-shifted directions of DPVSs for the polarization order *m* < 0, by relative phase difference analyses of either initial polarization angle offset Δ*φ*_0_ or relative polarizer angle Δ*θ*. (**c**) Accuracy enhancement of DPVSs via amplified *f_DPVSs_* with the combination of polarization order *m* and OAM order *ℓ*.

**Supplementary Note 4: Accuracy-enhanced Doppler shift measurement via DPVSs**

According to the definition of relative error, |(*Δf*_exp_-*Δf*_th_)/*Δf*_th_|×100%, the larger the theoretical Doppler shift *Δf*_th_ the smaller the relative error for a same measurement system, and thus the higher the measurement accuracy. Hence, amplifying Doppler shift can be viewed as an effective route to achieve accuracy-enhanced Doppler-shifted measurement. Figure S4 quantitatively illustrates accuracy-enhanced magnitudes for Doppler shift measurement of DPVSs, according to Eq. (3) in main manuscript. Here, we set the absolute error ratios, *κ_1_* and *κ_2_*, from 0.6 to 1.4, as the absolute errors of DPVSs, DS and DPS measurement are basically in an order of magnitude. Considering the modulated capabilities of SLM and VR, the simulated polarization orders and OAM orders are respectively set at: *ℓ* = 1 ~ 30, |*m|* = 1 ~ 20. From Fig. S4, **the larger the absolute error ratio, the more salient the accuracy-enhanced effect**. Furthermore, when the polarization order, |*m|,* is much more larger, and the OAM order, *ℓ*, is more smaller, the accuracy-enhanced ratio, *N_1_*, of DPVSs versus DS is more salient, whereas one of DPVSs versus DPS, *N_2_*, is more less obvious. The reverse things are displayed on the accuracy-enhanced ratio, *N_2_*, of DPVSs versus DPS. That is, **the larger the difference of |*m*| and *ℓ*, the more salient the accuracy-enhanced effect**. Despite distinct accuracy-enhanced effect, all accuracy-enhanced ratios: *N_1_* > 0 and *N_2_* > 0, regardless of |*m|* and *ℓ*. Particularly, the maximum accuracy-enhanced ratios *N_1_* ≈ 30 and *N_2_* ≈ 45, when *κ_1_* = *κ_2_* = 1.4. If the absolute error ratios, *κ*, and/or the difference of |*m|* and *ℓ* can be set as much more larger, the accuracy-enhanced effect will be more salient. These simulated results are well consistent with theoretical analyses in Eq. (3).


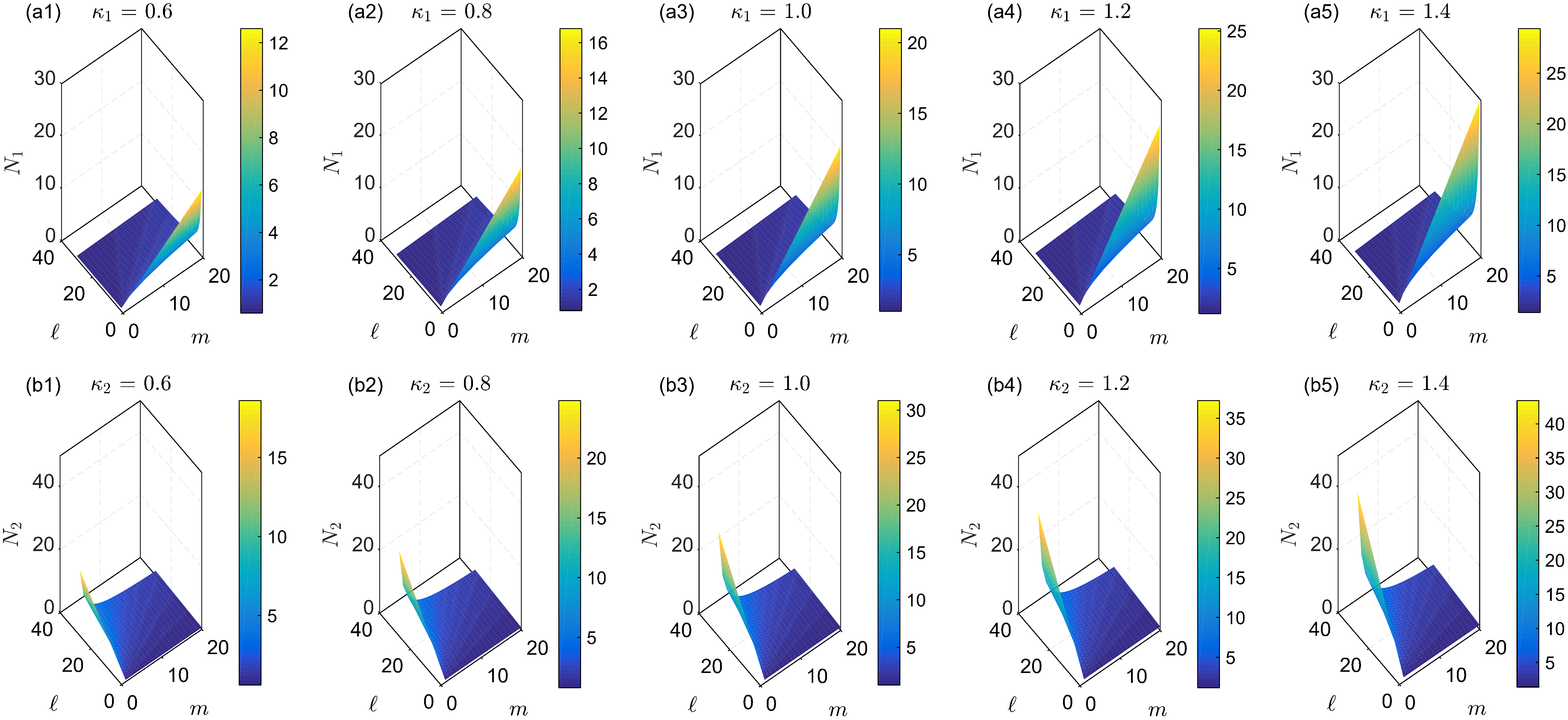


**Supplementary Fig. S4 Numerically simulated accuracy enhancement of DPVSs versus DS (a1-a3) and of DPVSs versus DPS (b1-b3) according to Eq. (3).**

**Supplementary Note 5: Signal post-processing for observing GDSs under uniformly varying velocity**

To extract GDSs when the target’s velocity varies uniformly, we implement signal post-processing following the four-step procedures after the detection of the GDSs. As shown in Fig. S7, we address original GDS of *I₁* recorded with the polarized angle of LP3 at 0° and *m* = 8, *ℓ₁=−ℓ₂*=5 as well as sampling points 2500 over 0.1 s, as an example for illustration.

(ⅰ) In the first step, we implement discrete STFT to achieve time-frequency analysis for collected non-stationary GDS, where the frequency spectrum can thus be given as:

$S\left[t,f\right] = \sum_{n=0}^{N-1} I_{1}\left[n\right]w\left[n-t\right]\exp^{-j2\pi fn/N}$ (S14)

where *w*[·] represents a Hanning window of width *N* =512 to suppress spectrum leakage. Note that the longer the window width, the higher the frequency resolution and the lower the temporal resolution, and vice versa. *t* and *f* are time and frequency vectors, respectively. *n* denotes the overlap point numbers given by the overlap ratio of 0.9. Within the real calculations, an original signal is first divided by the window width N into several frames, and thus the sliding step size can be given by *N* − *n*. Then, we implement the windowing treatment and FFT calculations with point numbers of 1024, where the frequency spectra for the start and end signals are distorted, and hence we delete these components.

(ⅱ) In the second step, since generalized Doppler spectrum embraces multiple frequency spectrum peaks with distinct power magnitudes, we fail to use the traditional maximum method to extract the signal skeletons. To address this problem, we provide a threshold binarization strategy to maintain multiple frequency peaks within STFT spectrum *S*. Specifically, we set a threshold *T* = −27 dB to subsequently binarize the pixel amplitudes of *S*. This process can be given by:

$S(x,y)\text{=}\left\{\begin{matrix} 1, S(x,y)>T \\ 0, otherwise \end{matrix}\right.$ (S15)

Here, the white regions (1) represent signals, while the black regions (0) give the background. The threshold *T* can be adaptively selected by taking the average of *S*(*x*, *y*) and then altering the offset according to the practical images. This step would provide reliable input for subsequent feature extraction.

(ⅲ) In the third step, we first perform the region scanning, where we find all consistent '1' regions for each time column vector. Assuming that *M* regions can be found, where the start and end indexes of each region can be given as *sⱼ* and *eⱼ*, respectively, *j* = 1, 2, ..., *M*, where *M* = 39 being time channels. Then, we can filter out a certain width based on the region width *wⱼ* = *eⱼ − sⱼ* + 1, and thus maintain valid regions. Subsequently, we calculate the centroid frequency *fⱼ* for each valid region according to:

$f_{j} = \frac{1}{w_{j}}\sum_{k=s_{j}}^{e_{j}} f_{k}$ (S16)

where *fₖ* is the *k*-*th* frequency value in the frequency axis. Furthermore, we arrange the centroid frequency in descending order within each time channel. Lastly, we can index all time channels according to this law to acquire four skeletons of generalized Doppler spectrum.

(ⅳ) In the last step, in order to reflect local trends, we use a segmented linear interpolation method to fit and smooth the four centroid curves, respectively. Hence, we can estimate the frequency values with variable time points. Eventually, we obtain equations of linear regressions according to the fitting curves, segmentally.


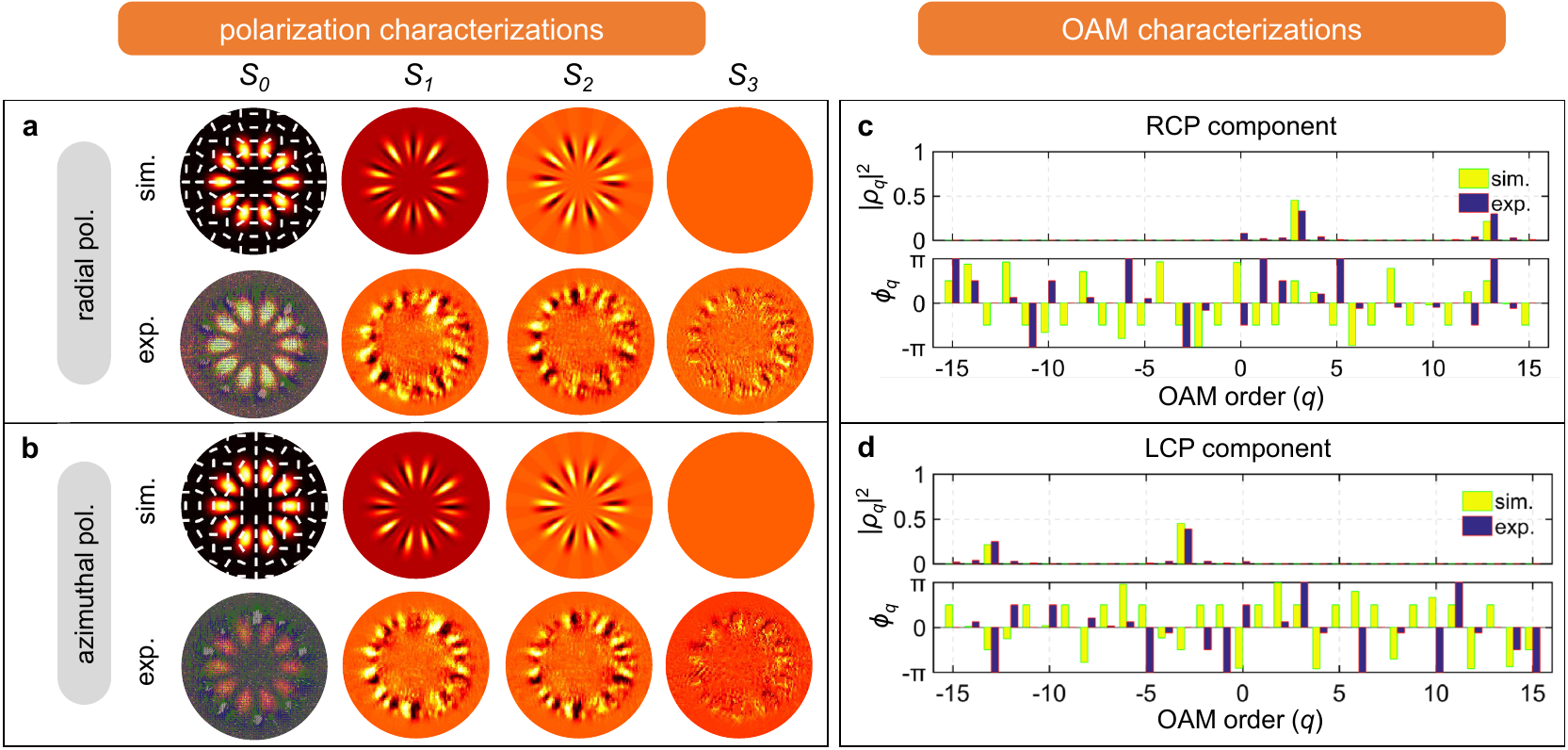


**Supplementary Fig. S5 Simulated and experimental characterizations of polarization and OAM properties for generated VPDVFs with *m*=8, *ℓ₁=-ℓ₂*=5.** (a) and (b) Polarization characterizations by Stokes parameters for radially *φ_0_*=0 and azimuthally φ*_0_*=*π*/2 polarized VPDVFs, respectively. (c) and (d) OAM characterizations via OAM power spectrum and OAM phase spectrum for two orthogonal circularly polarized components of radially or azimuthally polarized VPDVF.


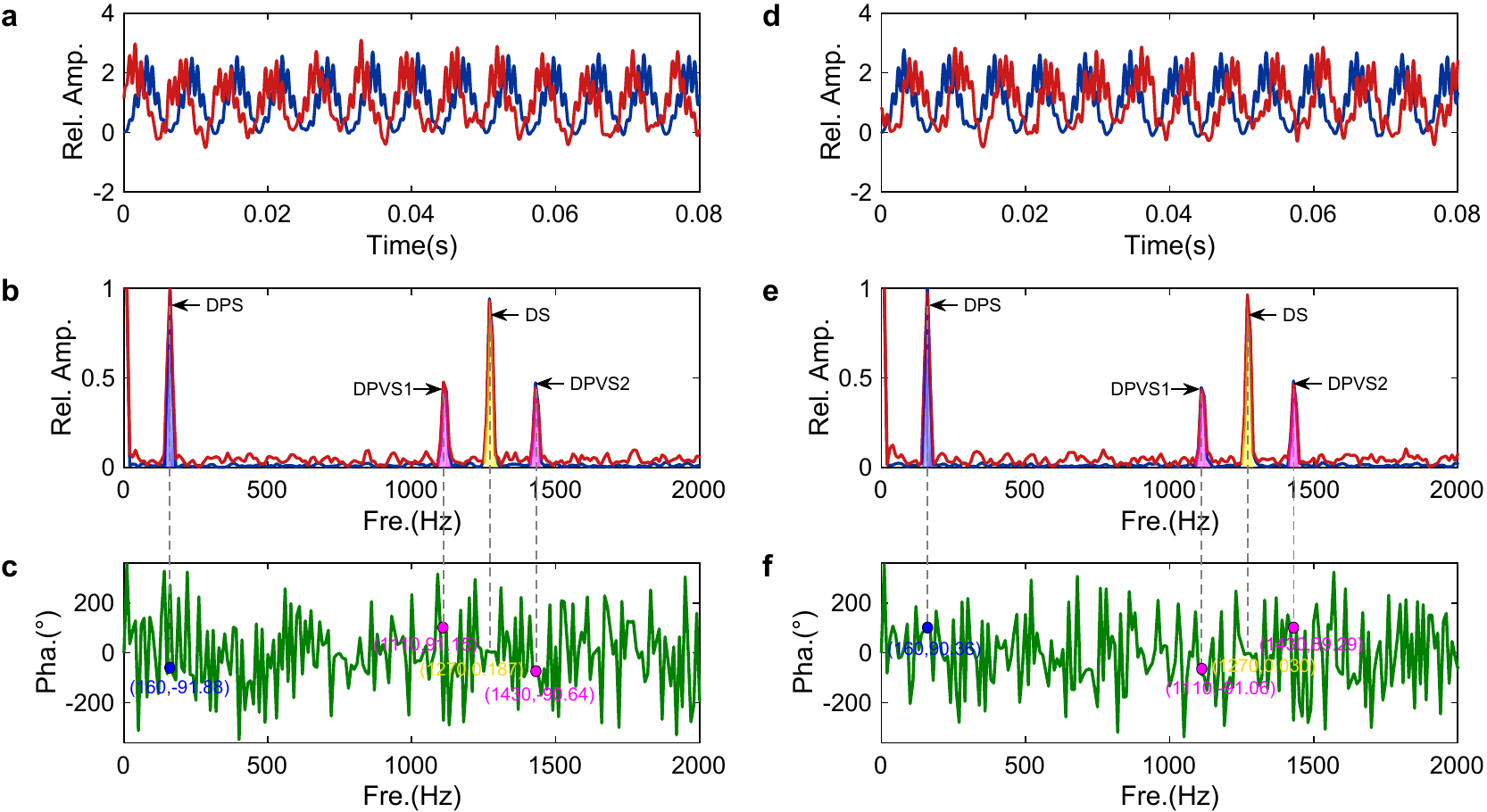


**Supplementary Fig. S6 Experimentally measured GDSs and their Doppler spectra** (a) and (d) Measured GDSs through polarizers at angles of 0° and 15° for radially polarized VPDVF with m=1, *φ*_0_=0, ℓ₁=-ℓ₂=8, respectively. (b) and (e) Measured generalized Doppler amplitude spectra, correspondingly. (a) and (d) Measured relative phase difference between GDSs through polarizers at angles of 0° and 15°.


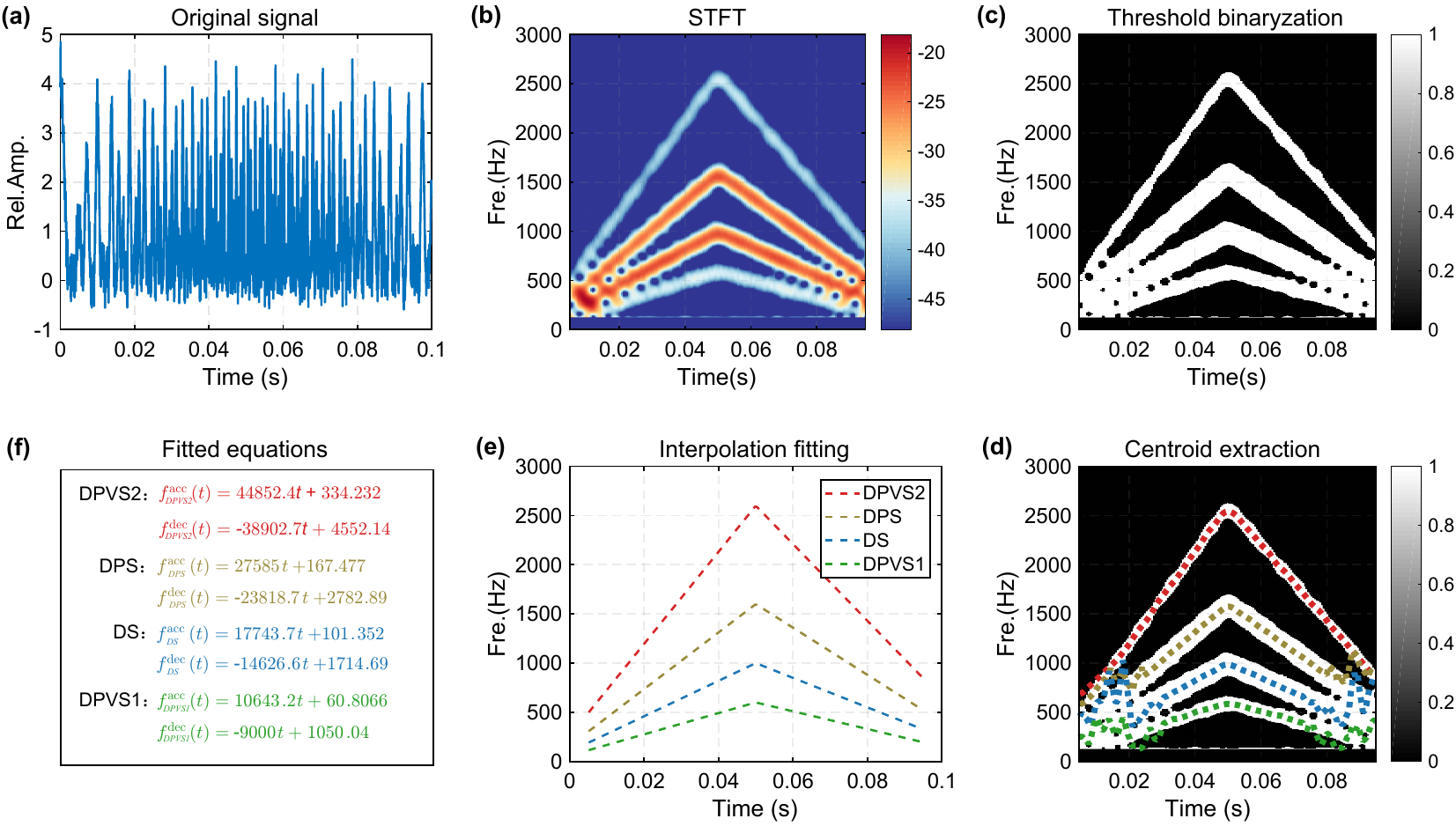


**Supplementary Fig. S7 Signal post-processing for GDSs with uniformly variable velocity.** This process including several steps: STFT, threshold binaryzation, centroid extraction, interpolation fitting as well as equation fitting.

**Supplementary references:**

1. McLaren, M., Konrad, T., & Forbes, A. Measuring the nonseparability of vector vortex beams. *Phys. Rev. A* 92(2), 023833 (2015).
2. Shen, Y., Nape, I., Yang, X., Fu, X., Gong, M., Naidoo, D., & Forbes, A. Creation and control of high-dimensional multi-partite classically entangled light. *Light Sci. Appl.* 10(1), 50 (2021).
3. Yao-Li, Y. L., Hu, X. B., Perez-Garcia, B., Bo-Zhao, B. Z., Gao, W., Zhu, Z. H., & Rosales-Guzmán, C. Classically entangled ince–gaussian modes. *Appl. Phys. Lett.* 116(22) (2020).
4. L. Fang, Z. Wan, A. Forbes, and J. Wang, Vectorial Doppler metrology, *Nat. Commun.* 12, 1 (2021).
5. Z. Wan, L. Fang, and J. Wang, Direction-discriminated rotational Doppler velocimetry with circular polarized vortex beams, *Opt. Lett.* 47, 1021 (2022).
6. Z. Wan, Y. Liang, X. Zhang, Z. Tang, L. Fang, Z. Ma, S. Ramachandran, and J. Wang, Remote Measurement of the Angular Velocity Vector Based on Vectorial Doppler Effect Using Air-Core Optical Fiber, *Research* 2022 (2022).
